# Urethane-Foam-Embedded Silicon Pressure Sensors including Stress-Concentration Packaging Structure for Driver Posture Monitoring

**DOI:** 10.3390/s22124495

**Published:** 2022-06-14

**Authors:** Seiichi Takamatsu, Suguru Sato, Toshihiro Itoh

**Affiliations:** 1 Department of Precision Engineering, School of Engineering, The University of Tokyo, Hongo 7-3-1, Bunkyo-ku, Tokyo 113-8656, Japan; itoh@pe.t.u-tokyo.ac.jp; 2 Graduate School of Frontier Sciences, The University of Tokyo, Kashiwano-ha 5-1-5, Kashiwa 277-8563, Japan; 2061512978@edu.k.u-tokyo.ac.jp

**Keywords:** automobile driver monitoring, hyper elastic foam, stress transmission structure, sensor array, silicon pressure sensor

## Abstract

We propose urethane-foam-embedded silicon pressure sensors, including a stress-concentration packaging structure, for integration into a car seat to monitor the driver’s cognitive state, posture, and driving behavior. The technical challenges of embedding silicon pressure sensors in urethane foam are low sensitivity due to stress dispersion of the urethane foam and non-linear sensor response caused by the non-uniform deformation of the foam. Thus, the proposed package structure includes a cover to concentrate the force applied over the urethane foam and frame to eliminate this non-linear stress because the outer edge of the cover receives large non-linear stress concentration caused by the geometric non-linearity of the uneven height of the sensor package and ground substrate. With this package structure, the pressure sensitivity of the sensors ranges from 0 to 10 kPa. The sensors also have high linearity with a root mean squared error of 0.049 N in the linear regression of the relationship between applied pressure and sensor output, and the optimal frame width is more than 2 mm. Finally, a prototype 3 × 3 sensor array included in the proposed package structure detects body movements, which will enable the development of sensor-integrated car seats.

## 1. Introduction

Driver-monitoring sensors that detect the health and cognitive status of car drivers have gained much attention for future automotive safety technology, enabling cars to request emergency responses up to level 3 automated driving and to give an alert to drivers who are distracted, fatigued, and drowsy [1,2,3,4,5,6,7,8]. Methods for monitoring driver inattentive states that lead to road accidents [2,9,10,11] involve detecting driver behavior with cameras [6,11,12,13], detecting driver fatigue and drowsiness with vitals sensors [6,7,14,15], and measuring body pressure distribution with pressure-sensor matrices in the seats [16,17,18]. Driver monitoring must meet the following requirements for commercial applications [2,6,9]:

1. Avoid discomfort to the driver;
2. Avoid interference with cognition, judgment, and automobile operation.

The main method in commercial applications is a non-contact method that uses a camera to detect the driver’s face and body movements [13]. However, there is much information that cannot be obtained with cameras due to changes in brightness inside the car, ambient light, and the driver’s clothing [19]. Therefore, a method of measuring pressure distribution by integrating pressure sensors [16,18] into car seats, which have the advantage of direct contact with the body, is attracting attention when used in combination with a camera.

Previous studies [16,17,20,21] reported that it is theoretically possible to estimate driver distraction and fatigue levels by measuring body pressure distribution to detect driver posture and behavior. Driver posture was reported to be correlated with driving comfort, and a decrease in comfort could result in driver distraction [17]. There is also a correlation between driver wakefulness and fatigue levels and driving posture. Drivers were found to frequently re-sit or tilt back and forth when drowsiness and fatigue levels increased [22,23]. However, current pressure sensors include a combination of electrodes on a flexible printed circuit board (PCB) or other plastic, rubber, or fabric substrate for detecting capacitance change under pressure [21,23,24]. This type of pressure sensor is stiffer than urethane foam and cannot deform to follow the shape of the human hip, giving the user a sense of discomfort when sitting. In addition, since the rubber-type sensors are not stable for long time use, sensors made of inorganic materials, such as silicon, which are stable over the long term, are preferable. Thus, new comfortable and long-term stable pressure sensors that can be incorporated into a car seat are necessary for the commercialization of car-seat-type driver-monitoring technologies.

Since micro-electromechanical systems (MEMS)-based silicon diaphragm-based pressure sensors have become widespread, highly sensitive (1–10 N), and low cost, we measure body pressure distribution using an array of commercially available silicon pressure sensors under a urethane foam. The specifications required for such sensors are sensitivity ranging from 0 to 10 kPa to detect driver movements on a seat and high linearity of the sensor-output characteristics. However, silicon is rigid and hard, with a Young’s modulus of 193 GPa, compared with urethane foam with a Yung’s modulus of 0.06 GPa. Therefore, since the compressive pressure over urethane foam is difficult to transmit to silicon pressure sensors, one challenge is to develop a package structure to concentrate pressure from the urethane foam and transmit that pressure to silicon pressure sensors [25,26]. The sensor output is not linear to the applied pressure because urethane foam is easily deformed, and pressure applied over the foam is dispersed not only to the sensor but also to ground substrates and other components. Thus, another challenge is to improve the linearity of the sensor output. The deformation characteristics of foam, such as urethane foam, are different from rubber, soft plastic, or other electronics packaging materials. Rubber materials, for example, monotonically increase in compressive stress as compressive strain is applied. Urethane foam is a series of cells that are individually filled with air [25,26]. When compressed, the foam is divided into three regions: the linear elastic region, where stress increases linearly with strain due to bending deformation of the cell walls; the plateau region, where deformation proceeds at a nearly constant stress due to buckling of the cell walls; and the densification region, where the cells are crushed, and compressive stress rapidly increases. Since the plateau region, which deforms significantly at a certain stress, has the effect of dispersing forces on the uneven bodies of silicon pressure sensors and ground substrate, the strain on the silicon sensor, especially on its cover, is greatly distorted, while the amount of strain in the surrounding substrate area is smaller. This difference causes a large stress to fill the difference in the outer edge of the covers of silicon pressure sensors, resulting in non-linearity. A specific package structure is needed to prevent this geometric non-linearity of sensors.

**Figure 1:**
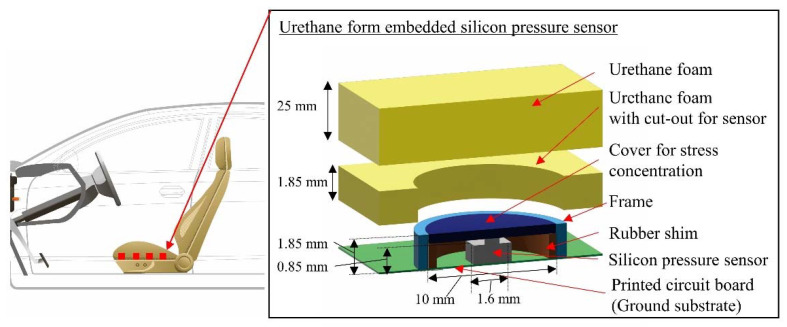
Concept and details of proposed package structure. Silicon pressure sensors are placed on PCB, and cover is supported by rubber shim surrounded with frame. Urethane foam with cut-outs for sensors is placed around the sensors, and additional urethane foam without cut-outs is placed over the sensors.

The main goal of our research is to develop a pressure-sensor array with a package structure that can function under urethane foam for driver monitoring. Since pressure dispersion and output non-linearity of silicon pressure sensors are problematic under urethane foam, we propose a pressure-sensor package structure with a cover that concentrates pressure and a frame that prevents non-linear stress. This is because we found that non-linear stresses are concentrated in the outer edge of the cover. Figure 1 shows the details of the proposed package structure, which consists of silicon pressure sensors wired and mounted on a PCB, which is a ground substrate, a rubber shim, cover for pressure concentration, and frame. Urethane foam with cut-outs is placed around the silicon pressure sensors, and an additional urethane foam without cut-outs is placed over the sensors. In this study, the MEMS pressure sensor of HSFPAR003A, ALPS ALPINE 136 CO., LTD, is used as a pressure sensor that can directly measure 1~10 kPa of human body pressure. Most MEMS pressure sensors are mounted in a package with an air hole to measure air pressure, and its sensitivity is about 10 k~100 kPa, while this sensor receives 1~10 N force with a silicon diaphragm structure, and its sensitivity with several-centimeter package structure is several kPa, which is not suitable for measuring human body pressure distribution of 1~10 kPa.

We conducted experiments and simulations to evaluate the proposed package structure, and the results indicate the effectiveness of the cover regarding stress concentration and the frame regarding non-linearity reduction. The width of the frame was adjusted and optimized. Finally, a prototype 3 × 3 array of silicon pressure sensors was constructed and integrated into the proposed package structure to verify the feasibility of driver monitoring.

## 2. Materials and Methods

### 2.1. Simulations

**Figure 2:**
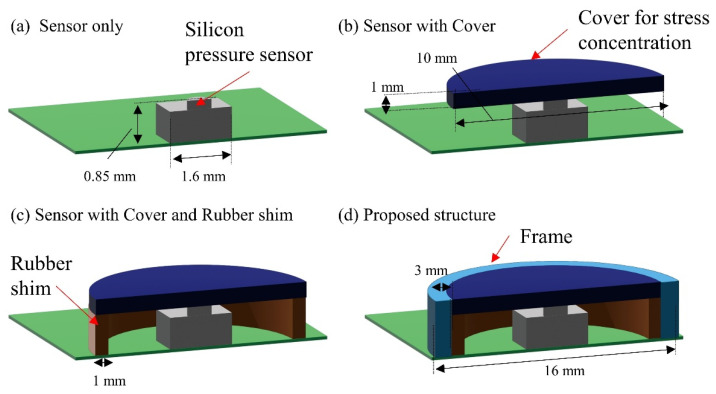
Four different types of package structures: (**a**) silicon pressure sensors only; (**b**) silicon pressure sensors with pressure-concentration cover; (**c**) silicon pressure sensors with cover and rubber shim; (**d**) silicon pressure sensors with proposed package structure consisting of cover, rubber shim, and frame.

Simulations were conducted on the four different types of package structures shown in Figure 2 using the finite element method (FEM) simulation software Abaqus (Dassault Systems, Tokyo, Japan). These package structures were sensors only, sensors with cover, sensor with cover and rubber shim, and proposed structure with cover, shim, and frame. The four package structures in Figure 2 were modeled in three-dimensional CAD (AutoCAD, Autodesk, San Rafael, CA, USA). A polyurethane sponge was placed on top of the package, and 1–10 KPa, which is equivalent to body pressure, was applied from above for simulation.

However, the stress–strain characteristics of urethane foam are non-linear and not available in the FEN software library. Therefore, a mechanical model of polyurethane foam (density 65 kg/m^3^) taken from a car seat (AQUA, Toyota Motor Corporation, Toyota, Japan) was constructed by conducting uniaxial compressive stress and uniaxial tensile tests. A maximum-load 200 N force gauge and round flat compression table (CP-U-80, Aikoh Engineering, Osaka, Japan) were mounted on a bench-top load-testing machine (FTN-3001, Aikoh Engineering). Urethane foam was compressed by 70% in the bench-top load-testing machine to measure its stress–strain characteristics. For the tensile test, after urethane foam was cut into a dumbbell-like No. 1 shape using a laser cutter for the specimen shape, and a flat chuck was mounted on the force gauge, tensile testing was carried out until the specimen elongated by 20%. The measured non-linear stress–strain data were curve fitted using an Ogden model [27,28] and used as the material parameters of urethane foam for FEM simulation. Since this model can accurately express non-linear deformation behavior of a urethane foam up to the high strain region, we used the following Ogden model.

(1) $$W=\overset{N}{\underset{i=1}{\sum }}\frac{2\mu_{i}}{\alpha_{i}^{2}}\left\{\lambda_{1}^{\alpha_{i}}+\lambda_{2}^{\alpha_{i}}+\lambda_{3}^{\alpha_{i}}-3\right\}+\frac{1}{\beta_{i}}\left(J_{el}^{-\alpha_{i}\beta_{i}}-1\right),\;$$

where *W* is the strain energy function of a foam, *λ*_1_, *λ*_2_, and *λ*_3_ are the principal stretch ratios, *N* is the order of the model, and $\mu_{i}$, $\alpha_{i}$, and $\beta_{i}$ are material parameters. The *W* is the free energy per unit volume generated in a material when it is deformed. It is expressed using *λ*_1_, *λ*_2_, and *λ*_3_. The $\mu_{i}$, $\alpha_{i}$, and $\beta_{i}$ were derived from the theoretical solution of the stresses and test results and used in the simulator. They are calculated by combining data from the uniaxial compression and uniaxial tensile tests to construct a foam material model.

For the silicon pressure sensors, the relationship between strain and pressure was first measured using a micro compression tester (MST-I HR, Shimazu Corporation, Kyoto, Japan). Since the thickness of the diaphragm of a silicon pressure sensor (HSFPAR003A, ALPS ALPINE Co., Ltd., Tokyo, Japan) is unknown, it was calculated using the beam fixed at both-ends-uniform continuous distributed load model using the obtained experimental compression test results. A silicon pressure sensor was modeled as a beam with a thickness of 25 μm and width of 360 μm. In addition, Young’s modulus and Poisson’s ratio pairs of the acrylic sensor cover and frame, silicon pressure sensors, and rubber shim were 5.0 GPa, 0.35; 165 GPa, 0.30; and 1.4 MPa, 0.49, respectively.

### 2.2. Experiments

#### 2.2.1. Proposed Package Structure and Sensor-Array Fabrication

The proposed package structure was fabricated using a 3D printer (AGILISTA-3200, Keyence Corporation, Osaka, Japan) to form the cover, rubber shim, and frame. The material used for the cover and frame was acrylic (AR-M2, Keyence Corporation, Japan), and the material used for the rubber shim was low-hardness silicone rubber (AR-G1L, Keyence Corporation, Japan). The cover was disk shaped with a thickness of 1 mm and a radius of 5 mm. The rubber shim was a circular disk with a hole of an inner diameter of 8 mm, outer diameter of 10 mm, and height of 0.8 mm. The frame was circular with a hole of an inner diameter of 10 mm, outer diameter of 14 mm, and height of 1.8 mm. The silicon pressure sensors were soldered to a standard glass epoxy PCB substrate with a thickness of 1.6 mm. The sensor mounting and assembly were operated with conventional chip mounter (MRS-850RD, Okuhara Electric Corporation, Tokyo, Japan). The measured assembly error of the sensor chip was 40 μm in the height direction.

#### 2.2.2. Embedding in Urethane Foam

The urethane foam was rectangular with cut-outs for the sensors. The foam dimensions were 50 mm square and 25 mm high. Urethane foam (density: 65 kg/m^3^) was of a car seat (AQUA, Toyota Motor Corporation, Toyota, Japan), and the cut-outs were formed using a laser cutting machine VLS4.60 (Yokohama Systems Co., Ltd., Yokohama, Japan). The foam was placed around the sensors.

#### 2.2.3. Pressure-Sensor Evaluation Setup

A single pressure sensor embedded in the urethane foam was placed on the bench-top load-testing machine, and a load ranging from 0 to 10 kPa was applied to it to characterize it. The sensor contained a Wheatstone bridge of four piezoresistive strain gauges. A supply voltage of 3 V was applied from a DC precision power supply (PAR18-6A, TEXIO), and the output voltage of the Wheatstone bridge was measured using a source meter (2400 source meter, Keithley, Japan). We assumed a driver weighing 60 kg applies 10 kPa to a 30 cm × 20 cm seat equipped with the silicon pressure sensors and proposed package structure.

For the pressure sensors to monitor a drivers’ body pressure distribution, the measured voltage is converted to pressure using a 16 ch differential analog input board (NI9209, National Instruments, Tokyo, Japan) and LABVIEW software (National Instruments). The converted results are shown as voltage waveforms.

## 3. Results

### 3.1. Simulation and Experimental Results Comparing Four Types of Package Structures

**Figure 3:**
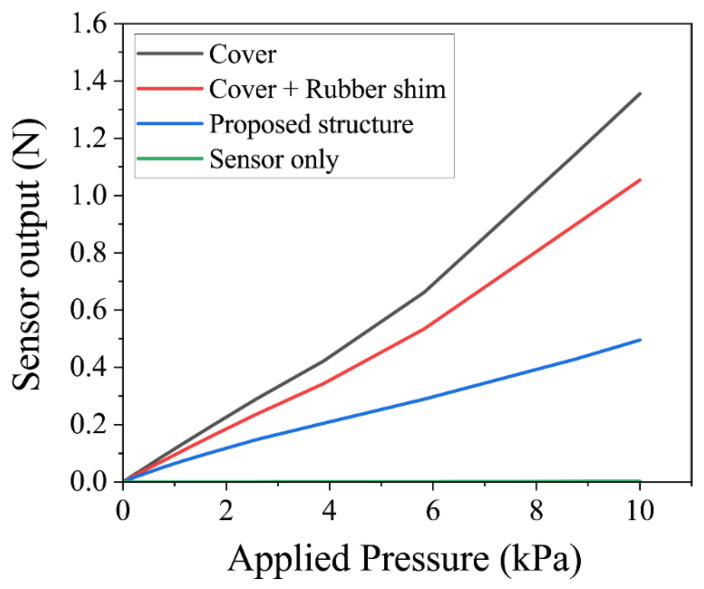
Simulation results of four different package structures.

**Figure 4:**
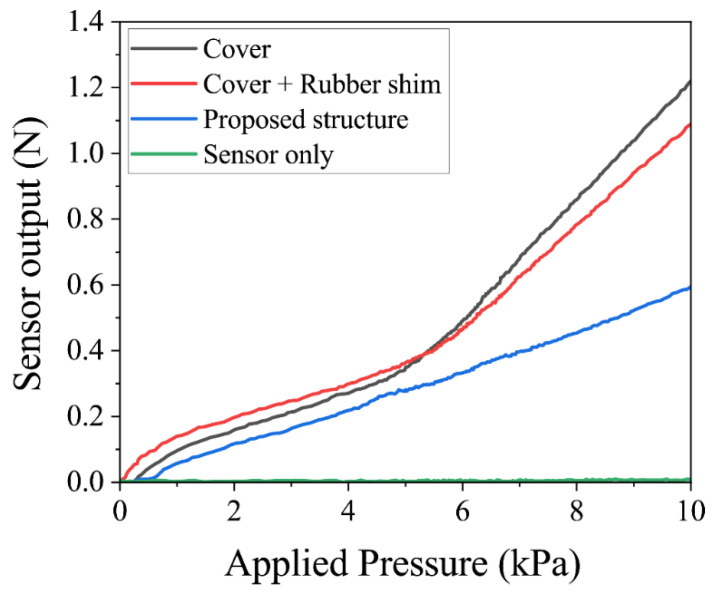
Experimental results of four different package structures.

The stress transmission of the applied load over the urethane foam to the silicon pressure sensors was evaluated through simulations, and the experiments for the four different package structures are shown in Figure 2. Figure 3 and Figure 4 show the simulation and experimental results, respectively.

When the urethane foam was placed directly on the silicon pressure sensors (Figure 2a), the stress was dispersed by the urethane foam, and sufficient force to deform the sensor was not applied. Since the stress was applied only to the small protuberances of the silicon sensor with a radius of 100 μm, the force applied to these protuberances was small, and the sensors did not deform, which can be seen in the sensor-only lines of Figure 3 and Figure 4.

When only a cover was attached to the silicon pressure sensors (Figure 2b), the transmitted force increased because the pressure was concentrated on the cover, and there was no rubber shim or frame. In the simulations, the sensor output was linear up to 5 kPa, above which the transmitted force to the sensor increased significantly. In the experiments, the effect of the urethane foam’s non-linearity was significant, and the amount of change in the stress increase was large, i.e., above 5 kPa.

**Figure 5:**
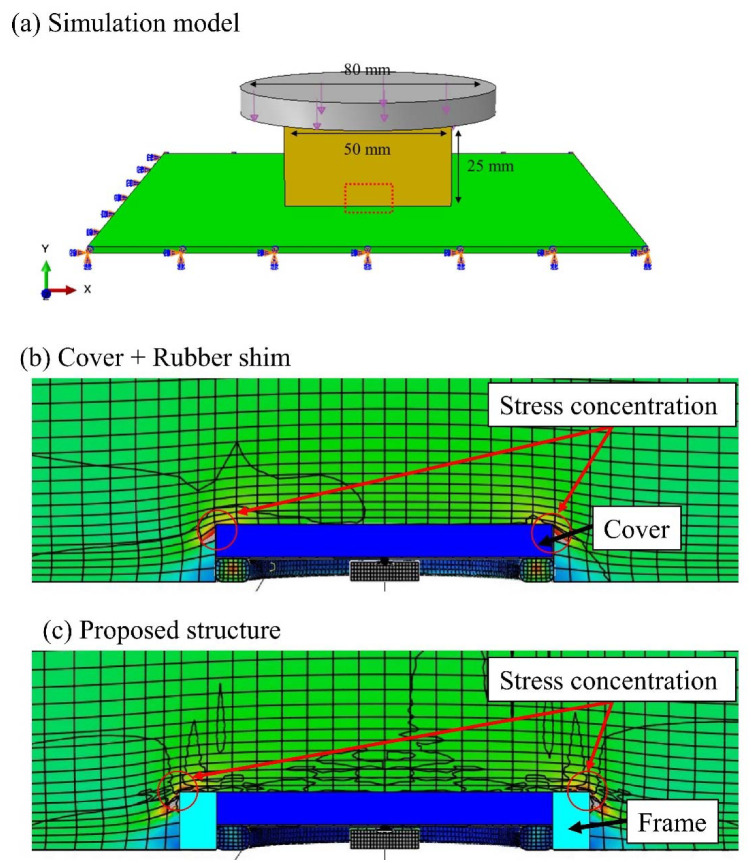
Simulation results of stresses applied to cover and frame. (**a**) The simulation result of the proposed package under body pressure applied. (**b**) The simulation result of a rubber shim and a circular cover package under body pressure applied. The large stress is concentrated on the edge of the cover. (**c**) The simulation result of proposed package consisting of a rubber shim, a circular cover, and a plastic frame structure. The large stress is concentrated not on the cover but on the plastic frame.

When the cover and rubber shim were attached to the silicon pressure sensors (Figure 2c), the stress transmission in this package structure increased similarly to that of the silicon pressure sensors with a cover. Basically, the package structure with the cover only was not stable compared with the package structure with cover and rubber shim because the cover was easily rotated, while the cover and rubber shim structure exhibited no rotation and exhibited stable output response. In the simulations and experiments, the sensor output was linear up to 5 kPa and increased sharply above 5 kPa. This is because if the urethane foam is compressed, the compressive displacement at the package cover of the silicon pressure sensors and that at the ground substrate differ, as shown in Figure 5. Then, the foam around the cover is prevented from deforming, and the large tensile stress is applied to the edge of the cover, resulting in the non-linear compressive force being applied to the cover of the package.

The proposed package structure, in which the frame receives the stress at the edge of the cover in other package structures, does not exhibit non-linearity above 5 kPa. To evaluate the linearity of pressure transmissions, a regression line was drawn on the plot of sensor output against applied pressure, and the root mean square error (RMSE) of the sensor output vs. the regression line was calculated. The results indicate that the proposed package structure had a high linearity with an RMSE of 0.049 N. The proposed package structure also shows that when the applied pressure between 0 and 10 kPa can be applied over the urethane foam and transmitted to the silicon pressure sensors, the sensors detect 0 to 0.6 N, which is their sensor range. Therefore, the proposed package structure can concentrate stress to the silicon pressure sensors, and the sensors can detect pressure with higher linearity.

### 3.2. Optimization of Frame-Structure Width

**Figure 6:**
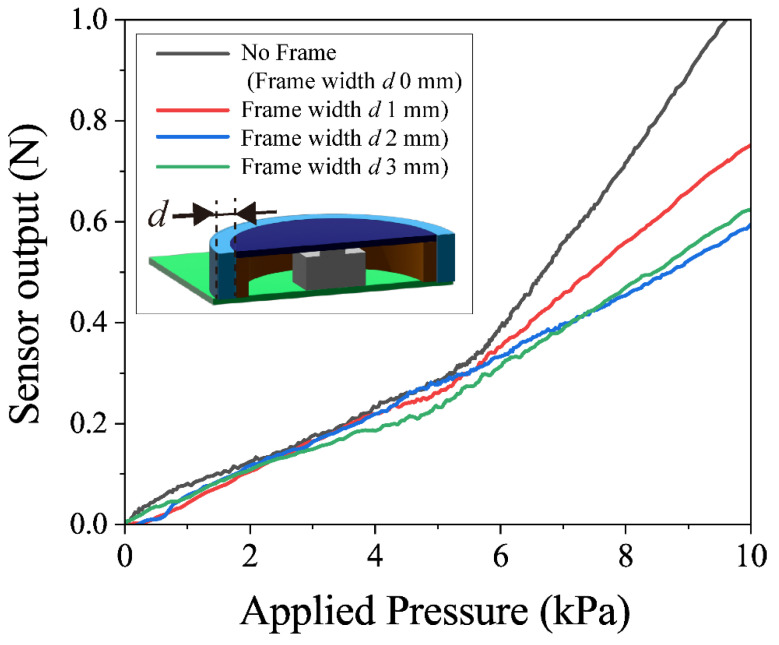
Relationship between applied pressure and sensor output of pressure sensors with different frame widths.

Non-linear tensile force from the urethane foam was applied in the area from the edge of the cover within 1 mm, causing the sensor response to be non-linear, as shown in Figure 5a. Therefore, the linearity of the sensor can be improved by attaching a frame surrounding the cover that does not transfer this tensile force to the cover of the sensors. To optimize the width of the frame, silicon pressure sensors with frames of varying widths, varying from no frame to 1, 2, and 3 mm, were fabricated, and their pressure sensitivities were evaluated, as shown in Figure 6. The experimental results indicate that the wider the frame, the better the linearity. As shown in Figure 6, to achieve high linearity over 5 kPa load, the frame width should be more than 2 mm. On the other hand, with no frame and frame widths of 1 mm, the sensor output increased rapidly over 5 kPa.

### 3.3. Driver Monitoring Demonstration Using Prototype Pressure Sensor Array with Proposed Package

**Figure 7:**
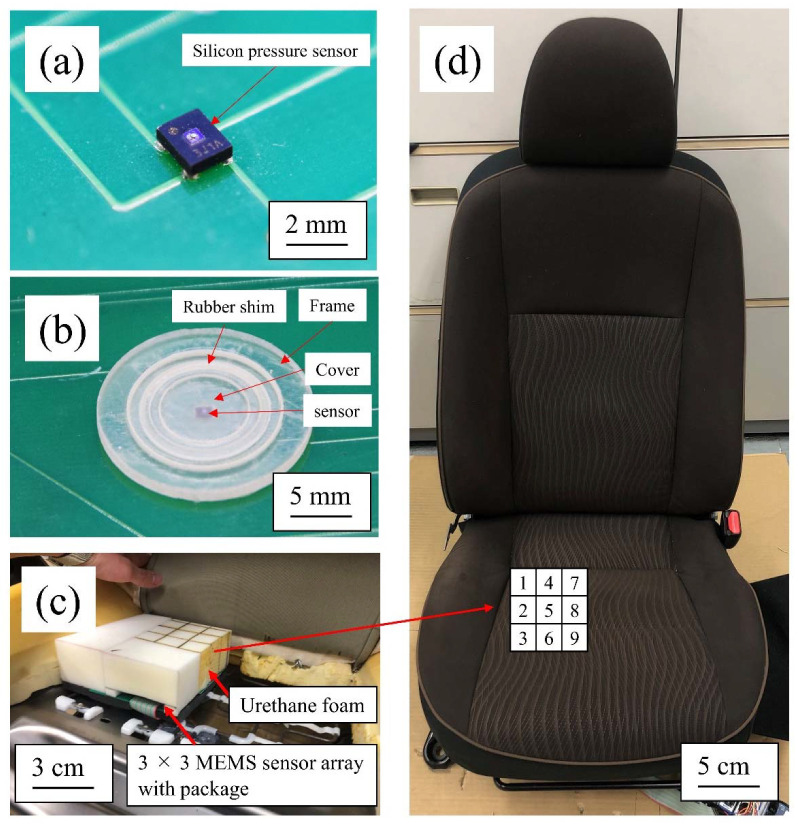
Prototype sensor array and its integration into car seat: (**a**) silicon pressure sensor on PCB; (**b**) package structure with rubber shim, cover, and frame; (**c**) urethane-foam-embedded silicon pressure sensor with proposed package structure; (**d**) car seat with silicon pressure sensor array and proposed package structure and array layout in car seat.

Figure 7 shows a photograph of the prototype 3 × 3 silicon pressure sensor array with the proposed package structure. Figure 7a shows a silicon pressure sensor mounted on a rigid PCB. The 3D printed rubber shim, cover, and frame were attached to the array, as shown in Figure 7b. Then, the urethane foam was removed from the car seat, and the sensor array with the proposed package structure was installed. The urethane foam with the cut-out sensor area was attached on top of the sensor array (Figure 7c). Finally, the seat fabric cover was placed on the urethane foam, as shown in Figure 7d. The sensors were calibrated prior to demonstration by measuring the sensor output of each sensor when a 10 kPa compressive load was applied using a 30 mm square jig attached to the bench-top load-testing machine. The sensor array was placed under the driver’s sciatic bone on the right side of the buttock.

**Figure 8:**
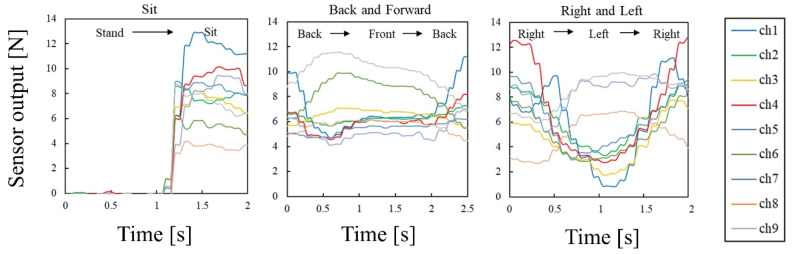
Output voltages of urethane-foam-embedded sensor array for three types of driver movements, such as sit, back and forward, and swaying the body right and left.

On the car seat with the prototype pressure sensor array, a driver sat down and performed three types of movements: sitting on the seat, swaying the body left and right, and swaying the body back and forth. Figure 8 shows the output voltages of the nine sensors in the pressure sensor array. The sensor output corresponding to the three types of movements is also indicated in Figure 8. The three different movements were measured several times, and the body pressure distribution for each movement was evaluated to see if the same posture could be determined. Table 1 shows the correlation coefficients calculated between the body pressure distributions in the same movements. The calculated correlation coefficients of three types of movements were approximately 0.9, indicating a strong positive correlation between body pressure distributions in the same movement. This suggests that the measured body pressure distribution contains information on posture and movement and that the proposed package structure is effective for driver monitoring.

## 4. Discussion

The problems with integrating silicon pressure sensors under the urethane foam in car seats are the pressure dispersion and output non-linearity of the sensors. Thus, we proposed a package structure that concentrates pressure. We conducted simulations and experiments to evaluate the effectiveness of our package structure and found that non-linear stresses are concentrated in the outer edge of the cover, since our package structure includes a frame that prevents these stresses. With the proposed package, urethane-foam-embedded silicon pressure sensors exhibited sensitivity to the applied pressure of 0–10 kPa and high linearity, since an RMSE of the sensor output vs. the regression line was 0.049 N. Experiments also showed that the width of the frame to ensure linearity should be at least 2 mm. The pressure sensor in this study responds to load changes of a few hertz. The sensor can sustain driver pressure of 0~10 kPa.

Finally, a prototype 3 × 3 silicon pressure sensor array included in the proposed packages was installed under a car seat to measure changes in body pressure distribution during three types of movements: sitting on the seat, swaying the body left and right, and swaying the body back and forth. The correlation coefficients between the body pressure distributions in the same movements were 0.9, indicating a strong positive correlation. Therefore, the pressure sensor array with the proposed package structure is expected to be applied to car seats for automatic driving. For the future work, the accuracy of the model to distinguish changes in a person’s posture should be improved by increasing the number of testing drivers. On the other hand, polyurethane foam is a component of chairs, sofas, and beds. Therefore, it is expected that our sensor will be used for health monitoring in the home.

## Figures and Tables

**Table 1:** Correlation coefficients for body pressure distributions for three types of driver movements.

Sitting on the Seat	Swaying the Body Left and Right		Swaying the Body Back and Forth	
	Right	Left	Forward	Back
0.878	0.969	0.994	0.903	0.934

## Data Availability

The data that support the findings of this study are available from the corresponding author, S.T., upon reasonable request.
